# Future Directions in Quantitative SPECT-CT Evaluation of Cardiac Transthyretin Amyloidosis: Correlation with Clinical and Morphological Parameters

**DOI:** 10.3390/diagnostics15040482

**Published:** 2025-02-17

**Authors:** Mirela Gherghe, Mario-Demian Mutuleanu, Tatiana Lucia Suta, Liliana Micu, Adina Elena Stanciu, Sinziana-Octavia Ionescu, Ciprian Cirimbei, Diana Loreta Paun, Andreea Jercan, Sorina Nicoleta Badelita, Daniel Coriu

**Affiliations:** 1 Nuclear Medicine Department, University of Medicine and Pharmacy “Carol Davila”, 050474 Bucharest, Romania; 2 Nuclear Medicine Department, Institute of Oncology “Prof. Dr. Alexandru Trestioreanu”, 022328 Bucharest, Romania; 3 Carcinogenesis and Molecular Biology Department, Institute of Oncology “Prof. Dr. Alexandru Trestioreanu”, 022328 Bucharest, Romania; 4 General Surgery Department 10, “Carol Davila” University of Medicine and Pharmacy, 050474 Bucharest, Romania; 5 General Surgery and Surgical Oncology Department I, Bucharest Institute of Oncology “Al. Trestioreanu”, 022328 Bucharest, Romania; 6 Endocrinology Department, University of Medicine and Pharmacy “Carol Davila”, 050474 Bucharest, Romania; 7 Endocrinology Department, National Institute of Endocrinology “C.I. Parhon”, 011863 Bucharest, Romania; 8 Hematology Department, Fundeni Clinical Institute, 022322 Bucharest, Romania; 9 Hematology Department, University of Medicine and Pharmacy “Carol Davila”, 050474 Bucharest, Romania

**Keywords:** quantitative SPECT-CT, cardiac amyloidosis, ATTR, SUVmax, SUVmean

## Abstract

**Background**: ATTRv and ATTRwt cardiac amyloidosis (CA) are underrecognized causes of heart failure with preserved left ventricular ejection fraction. The diagnosis of CA remains challenging due to low diagnostic suspicion and clinical overlap with more common diseases. The aim of this study was to use [^99m^Tc]-PYP SPECT-CT to perform a volumetric evaluation of bone scintigraphy to overcome the limitations of current practices. **Methods**: A monocentric prospective study was conducted to evaluate a lot of 22 patients with a mean age of 52.86 ± 13.80 years, diagnosed with hereditary cardiac transthyretin amyloidosis (ATTR). **Results**: Correlations between the quantitative SPECT-CT, clinical data, and morphological parameters were performed, demonstrating moderate to strong correlation of SUVmaxMyocardium/SUVmaxBone to both ECG low voltage and EchoGLS, SUVmaxMyocardium/SUVmaxLiver to myocardial gadolinium kinetics with T1 mapping MRI, diastolic disfunction, sensory–motor polyneuropathy, and EchoGLS, SUVmaxMyocardium/SUVmeanBone with diastolic disfunction and sensory–motor polyneuropathy, as well as SUVmaxMyocardium/SUVmaxSoft tissue to S II, respectively. **Conclusions**: The moderate to strong correlations among advanced quantitative SPECT-CT metrics and clinical and paraclinical data create the premises to use these parameters for early diagnosis of cardiac ATTR. Further multicentric studies in a larger patient population are needed to validate the newly identified quantitative SPECT-CT parameters.

## 1. Introduction

Systemic amyloidosis is a group of diseases caused by the deposition of insoluble fibrils, known as amyloid fibrils, in the extracellular spaces of tissues [1]. The number of identified amyloid fibril proteins is now 42, 14 of which appear only as systemic deposits, 23 are seen exclusively in local amyloid, while 5 can appear as both types [2]. Extracellular deposition of insoluble amyloid fibrils in various tissues generates progressive organ damage, leading to organ failure. From this contingent, nine amyloidogenic proteins are known to accumulate in the myocardium, causing significant cardiac impairment. Accordingly, over 98% of currently diagnosed cardiac amyloidosis results from fibrils composed of monoclonal immunoglobulin light chains (AL) or transthyretin (ATTR), either in its hereditary (ATTRv) or acquired (ATTRwt) form [3].

The different phenotypes of cardiac amyloidosis present a significant heterogeneity in clinical evolution, prognosis, and treatment approach [4].

Untreated AL amyloidosis involves an accelerated unfavorable course, with median survival less than 6 months, while ATTRv amyloidosis follows a different clinical development, depending on the specific inherited mutation, with either cardiomyopathy and/or sensory–autonomic polyneuropathy [5].

ATTRwt cardiac amyloidosis appears to be fairly common, with a prevalence of 10% to 16% among elderly patients with heart failure or aortic stenosis; autopsy studies have found cardiac ATTR amyloid deposition in up to 25% of individuals over 80 years of age [6].

Currently, ATTRv and ATTRwt cardiac amyloidosis are underrecognized causes of heart failure with preserved left ventricular ejection fraction. The diagnosis of CA remains challenging due to low diagnostic suspicion by clinical overlap with more common diseases resulting in myocardial thickening (e.g., hypertension, chronic renal failure, hypertrophic cardiomyopathy, aortic stenosis) and poor implementation of the diagnostic algorithm.

Early diagnosis of ATTR CA has a great influence on the prognosis of patients due to the benefit of specific treatment.

The European Society of Cardiology (ESC) and the American Heart Association (AHA) have developed dedicated guidelines, clarifying the diagnostic algorithm as well as the treatment strategy. The initial diagnostic assessment of a patient with suspected cardiac amyloidosis involves a comprehensive examination; the main findings are represented by clinical heart failure (HF) symptoms and paraclinical data such as myocardial hypertrophy (>12 mm) discordance with low voltage on ECG, increased NT proBNP levels, low-flow, low-gradient aortic stenosis, myocardial granular sparkling, abnormal left ventricular global longitudinal change, and others, representing common settings in which clinical suspicion of amyloidosis should be raised [7,8].

In this context, nuclear medicine imaging by scintigraphy is an important tool, providing a rapid and non-invasive diagnosis of ATTR cardiac amyloidosis.

Various tracers, all regarded as being equally effective, have been used, including ^99m^ technetium-bisphosphonate derivatives [^99m^Tc] pyrophosphate (PYP), [^99m^Tc]-3,3-diphosphono1,2-propanodicarboxylic acid (DPD), and [^99m^Tc] hydroxymethylene-diphosphonate (HMPD). DPD scintigraphy detects ATTR with type A fibril composition, which is found in both ATTRwt and the vast majority of ATTRv mutations [9,10]. Currently, the data provided by the literature suggest that [^99m^Tc]-PYP scintigraphy can accurately diagnose cardiac transthyretin amyloidosis (ATTR) and predict survival for patients undergoing treatment for amyloidosis [11,12].

The aim of this study was to use [^99m^Tc]-PYP SPECT-CT to perform a volumetric evaluation of bone scintigraphy with single-photon emission computed tomography–computed tomography (SPECT-CT) as an objective and quantitative method to overcome the limitations of current practices.

## 2. Materials and Methods

### 2.1. Patient Population

This monocentric prospective study was conducted including a population of 22 patients with a mean age of 52.86 ± 13.80 years, diagnosed with hereditary cardiac transthyretin amyloidosis (ATTR), presenting Glu54Gln TTR mutation; the patients were evaluated using whole body bone scintigraphy (WBS) and single photon emission computed tomography–computed tomography (SPECT-CT) performed in the Department of Nuclear Medicine of the National Institute of Oncology “Prof. Dr. Alexandru Trestioreanu” Bucharest, Romania. This study was approved by the ethics committees of our institution.

The following were the exclusion criteria:

- Higher radiotracer activity in the left ventricular cavity than in the myocardium.
- Amyloidosis variants other than ATTR.

### 2.2. Clinical Parameters

Multiple parameters data were collected, aiming to evaluate the degree of correlations between clinical data and quantitative information from SPECT-CT studies, with relevant significance for the diagnosis of cardiac ATTR. The following clinical parameters were collected and included in the database of the study: NYHA class, NTproBNP, Troponin, presence/absence of low voltage on electrocardiogram (ECG), diastolic disfunction, sensory-motor polyneuropathy, global longitudinal strain by 2D speckle tracking echocardiography (EchoGLS), myocardial gadolinium kinetics with T1 mapping by measurement of the intrinsic T1 relaxation time of the myocardium on the magnetic resonance imaging scanner, the systemic immune-inflammation index (S II), calculated using the S II = platelets × neutrophils/lymphocytes formula and systemic inflammation response index (SIRI), calculated using the SIRI = monocyte × neutrophil/lymphocyte formula (Table 1).

### 2.3. Image Acquisition and Reconstruction

An intravenous injection of 673.61 ± 56.64 MBq [^99m^Tc]-PYP was administered to each patient; imaging acquisition included a WBS acquisition one hour after the administration and a SPECT-CT scan with the field of view centered on the thoracic region three hours post-injection.

The imaging studies were performed using a GE Discovery DR670 SPECT-CT scanner (General Electric Healthcare, Chicago, IL, USA), complying with the European Association of Nuclear Medicine (EANM) guidelines; thus, the SPECT-CT protocol had the following settings: 128 by 128 matrix size, step and shoot mode, and 60 steps with 20 s per step. For scatter correction, a dual-energy window with 140 KeV peak energy ± 10 KeV and 120 ± 5 KeV was chosen. The SPECT scan was completed by a low-dose CT performed with 120 kV and 30 mA, dose modulation enabled (GE Smart Scan). The raw CT images had a 3.75 mm slice thickness and were then reconstructed in 2.5 mm slices by applying soft tissue enhancement filters provided by the vendor software.

SPECT data were reconstructed using the Ordered Subset Expectation Maximization (OSEM) iterative reconstruction algorithm with 6 subsets and 10 iterations. The process included resolution recovery, scatter correction, and attenuation correction based on the attenuation correction linear coefficient map derived from the conversion of the CT data into SPECT reconstruction-algorithm-compatible information.

The inclusion of SPECT/CT imaging helps to reduce false positive and equivocal results, thereby enhancing the diagnostic accuracy of the test. By using this imaging method, spatial resolution is improved, allowing for precise localization of myocardial uptake and accurate differentiation from the blood pool activity, a common source of error [13].

### 2.4. Image Interpretation

**Figure 1:**
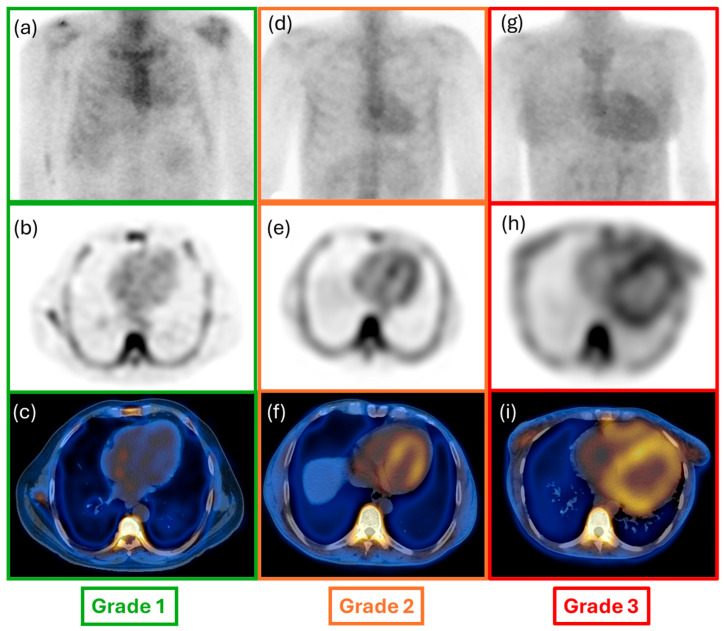
Whole-body and SPECT-CT axial view of a patient presenting cardiac ATTR: (**a**–**c**) Perugini Grade 1; (**d**–**f**) Perugini Grade 2; and (**g**–**i**) Perugini Grade 3.

**Figure 2:**
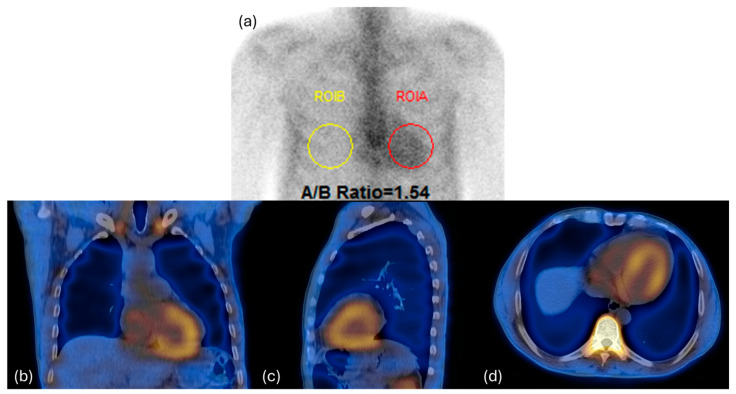
Whole-body and SPECT-CT acquisition of a 55-year-old patient presenting cardiac ATTR, Perugini Grade 2: (**a**) heart-to-contralateral ratio; (**b**) coronal SPECT-CT; (**c**) sagittal SPECT-CT; and (**d**) axial SPECT-CT.

The acquired images were independently reviewed using a dedicated workstation (GE Xeleris 5.0) by two nuclear medicine physicians with vast experience in nuclear cardiology. Following the qualitative and quantitative assessment of the WBS images, using the already established semiquantitative approach by Perugini et al., the cardiac uptake of each patient was graded from 0 to 3. Briefly, this system includes the following categories: Grade 0 indicates no cardiac uptake and normal bone uptake; Grade 1 signifies cardiac uptake that is less intense than bone signal; Grade 2 represents cardiac uptake with intensity similar to or greater than bone signal; lastly, Grade 3 denotes cardiac uptake with significantly attenuated or absent bone signal [14] (Figure 1 and Figure 2). For quantitative evaluation of planar imaging, the heart-to-contralateral ratio (H/CL) was performed.

**Figure 3:**
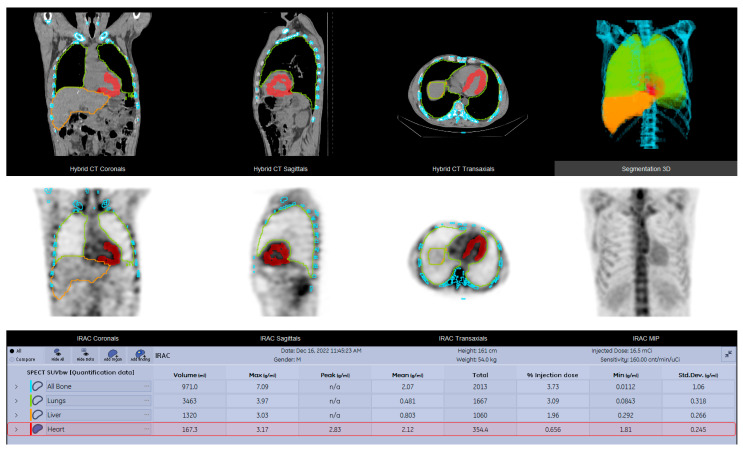
Contours of the VOIs automatically performed for the lungs, bone, and liver and semi-automatically for myocardium and soft tissue; high radiotracer uptake in the myocardium of the left ventricle.

For quantitative analysis of the SPECT-CT data, dedicated software (GE Q. Volumetrix MI) provided by the camera’s manufacturer was utilized for delineating the volume of interest (VOI). The contours of the VOIs were automatically established for lungs, bone, and liver and semi-automatically for myocardium and soft tissue (1.5 cm diameter sphere—shoulder muscle); all VOIs were manually adjusted whenever it was necessary (e.g., presence of degenerative bone lesions with high radiotracer uptake or improper organ edge delineation) (Figure 3). The preferred method for calculating radiotracer uptake was based on standardized uptake value (SUV) normalized to lean body mass (lbm). The following absolute SUVmax and SUVmean values were determined: SUVmaxMyocardium, SUVmaxLung, SUVmaxBone, SUVmaxSoft tissue, and SUVmaxLiver; SUVmeanMyocardium, SUVmeanLung, SUVmeanBone, SUVmeanSoft tissue, and SUVmeanLiver. Other SUV calculations were performed by computing the ratios for SUVmaxMyocardium to SUVmax and SUVmean of the reference organs (lungs, bone, liver, and soft tissue) (Table 2 and Table 3).

### 2.5. SUV Calculation

**Figure 4:**
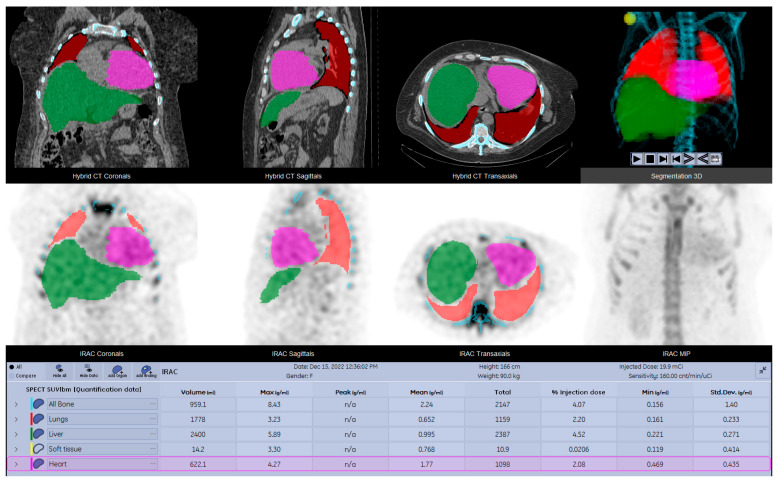
SPECT-CT data obtained with dedicated software; quantitative parameters for ratios, measurements, and objective image interpretation; bone, lungs, liver, soft tissue, and heart.

For the precise determination of SUV values, a range of parametric data were recorded for the purpose of activity correction. These parameters encompassed patient height, weight, gender, pre-injection activity, administered activity, post-injection activity, and the exact timing of measurements for the last three parameters. The decay for the injected dose and acquisition delay were automatically handled by dedicated software. Advanced segmentation tools with integrated AI capabilities were used for accurate delineation of VOIs, and the inherent precision of CT-based organ delineation allowed the quantification of SPECT statistics (Figure 4).

### 2.6. Scanner Calibration

To ensure accurate measurement in radiotracer uptake of the target lesions, dedicated scanner calibration needs to be performed. In our case, the SPECT-CT was calibrated using a uniform Jaszczak and a NEMA phantom, followed by the measurement of sensitivity values for both planar and SPECT acquisition.

### 2.7. Statistical Analysis

The data were analyzed using specialized statistical software, SPSS version 26.0. Because of the small sample of patients and the non-Gaussian distribution of data, non-parametric tests were used. Correlations between the quantitative SPECT-CT, clinical data, and morphological parameters were performed, and the strength of the correlation relationship was assessed using the Spearman rho correlation test; due to the small sample size and its limitations in identifying relevant confounding factors, we considered that this analysis, while relevant for evaluating correlations, should be reserved for studies with larger patient populations.

## 3. Results

All the variables included in the database were computed together for the statistical analysis of the degree of correlation; out of the entire dataset, only four clinical parameters presented statistically positive correlation coefficients in relation to the quantitative SPECT-CT parameters.

### 3.1. Correlation Between Presence/Absence of Low Voltage on ECG and Quantitative SPECT-CT Parameters

**Figure 5:**
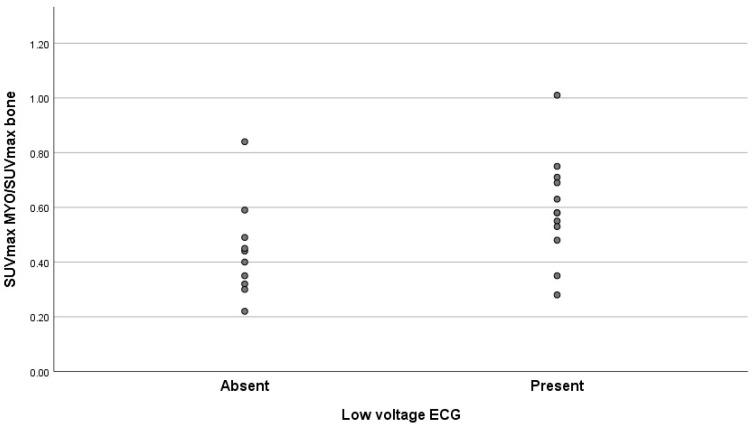
Presence/absence of low voltage on ECG and quantitative values of SUVmaxMyocardium/SUVmaxBone ratio.

The statistical analysis of the correlation grade between the presence/absence of low voltage on ECG and the quantitative ratio of SUVmaxMyocardium/SUVmaxBone indicated a moderate positive correlation with an index value of 0.425 and a *p* value equal to 0.049 (Table 4 and Figure 5).

The correlation between the two parameters represented in the figure above demonstrates that while most of the patients with low values of the SUVmaxMyocardium/SUVmaxBone ratio are clustered in the lower half of the scatterplot, the ones with high values of the same ratio are predominantly distributed in the upper half of the plot, thus corresponding to the positive correlation coefficient value.

### 3.2. Correlation Between S II and Quantitative SPECT-CT Parameters

**Figure 6:**
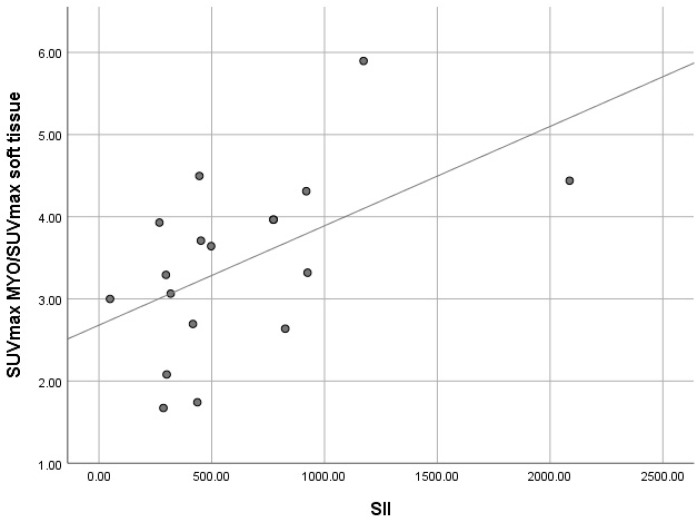
Graphical representation of the positive correlation between S II and SUVmaxMyocardium/SUVmaxSoft tissue.

The Spearman rho correlation test performed for the two variables S II and the ratio of SUVmaxMyocardium/SUVmaxSoft tissue resulted in a correlation coefficient of 0.556, indicating the presence of a relatively high degree of correlation between them (Table 5 and Figure 6).

By performing the scatterplot of the two variables, the SUVmaxMyocardium/SUVmaxSoft tissue ratio and S II, a clear correspondence between their high values can be observed suggesting the presence of the positive correlation.

### 3.3. Correlation Between Myocardial Gadolinium Kinetics with T1 Mapping and Quantitative SPECT-CT Parameters

The statistical evaluation of the correlation between the changes present in the variable myocardial gadolinium kinetics with T1 mapping MRI and the values of the SUVmaxMyocardium/SUVmaxLiver ratio showed a very high degree of positive correlation between the two parameters with a correlation index of 0.814 and a *p* value equal to 0.049 (Table 6).

### 3.4. Correlation Between Global Longitudinal Strain by 2D Speckle Tracking Echocardiography and Quantitative SPECT-CT Parameters

**Figure 7:**
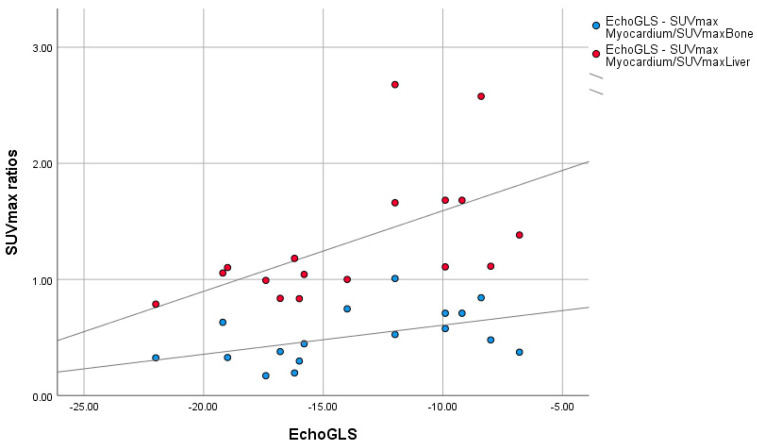
Scatterplot of SUVmax ratios distribution by EchoGLS.

In comparison to the results obtained from the statistical analysis of other variables included in this study, the Spearman rho test resulted in negative correlation coefficient indexes of 0.495 and 0.692 for the correlation assessment of EchoGLS and the SUVmaxMyocardium/SUVmaxBone ratio and EchoGLS and the SUVmaxMyocardium/SUVmaxLiver ratio, respectively (Table 7 and Figure 7).

The scatterplot represented in Figure 7 demonstrates the presence of a clear positive association between the EchoGLS values and the SUVmax ratios, especially for the SUVmaxMyocardium/SUVmaxLiver ratio.

### 3.5. Correlation Between Diastolic Disfunction and Quantitative SPECT/CT Parameter Ratios

The Spearman Rho analysis performed between the variable diastolic disfunction severity and quantitative SPECT-CT ratios has shown a significant moderate positive correlation between the degree of diastolic impairment and SUVmaxMyocardium/SUVmaxLiver with a correlation coefficient of 0.486 and SUVmaxMyocardium/SUVmeanBone with a correlation coefficient of 0.463, with statistically significant *p* values of 0.022 and 0.030, respectively (Table 8).

### 3.6. Correlation Between Sensory–Motor Neuropathy and Quantitative SPECT/CT Parameter Ratios

The statistical analysis of the correlation grade between the severity of sensory–motor neuropathy and quantitative ratios of SUVmaxMyocardium/SUVmaxLiver and SUVmaxMyocardium/SUVmeanBone indicated a moderate positive correlation with an index value of 0.666 for the first correlation and a *p* value equal to 0.001 and 0.450 for the second correlation and a *p* value equal to 0.036 (Table 9).

## 4. Discussion

In terms of diagnostic procedures, there are still a few challenges to be addressed when it comes to a timely and effective identification of the disease. Although endomyocardial biopsy is considered the gold standard for diagnosing ATTR, it has disadvantages such as: being invasive or inaccessible in most of the medical centers, all of this without providing important information regarding disease extent, prognosis or progression of disease [15,16,17,18]. Therefore, the elaboration of novel clinical practice guidelines have established a non-invasive diagnostic strategy for patients with cardiac amyloidosis based on the Perugini score, stating that a score of 2 or 3 and the absence of blood dyscrasia, represent enough evidence for TTR cardiac amyloidosis diagnosis [14].

The present study included 22 patients with CA, aiming to compare and correlate the data obtained using parameters quantitative obtained from planar and three-dimensional data with various clinical and paraclinical variables. The results indicate that quantitative SPECT-CT measurements performed using [^99m^Tc]-PYP can represent potential quantitative markers for early diagnosis and also correlated with different degrees of strength with different paraclinical diagnostic markers for ATTR CA: low voltage on ECG, global longitudinal deformation (GLS) obtained by echocardiography and T1 mapping by cardiac MRI examination, diastolic disfunction and sensory-motor neuropathy; all these indicators represent valuable diagnostic tools for cardiac amyloidosis evaluation [13,14].

Several quantitative parameters have been largely evaluated, the most relevant being represented by the SUV max and SUV-derived parameters calculated using the intensity of radiotracer uptake in different tissues such as: muscle, bone and blood, for the normalization of the myocardial uptake [19].

Recent efforts of the EANM (European Association of Nuclear Medicine) in developing quantitative SPECT-CT guidelines aiming to standardize the use of quantitative methods using high-performance SPECT gamma cameras, have enabled the emergence of new applications of the SPECT-based parameters [20]. However, the high degree of variation in the analyzed data resulting from the studies and the use of different software analysis programs have led to heterogeneous outcomes [19].

The only current consensus is represented by the optimal time to perform the SPECT-CT acquisition at 3 hours after the administration of the radiotracer, because of the decreased blood pool radiotracer uptake and higher lesion to background ratio, aiming to reduce the number of false positive results [21].

A study conducted by Costa et al. which included 17 patients with Perugini score of 1 (3 patients), 2 (4 patients), 3 (10 patients) demonstrated a strong negative correlation of the score with ventricular voltage/myocardial mass ratio (r = −0.70, *p* = 0.003), indicating that whenever high deposit of amyloid was present in the myocardium the intensity of the electrical signal was low [22]. These findings are consistent with the ones from our study in which high values of SUVmaxMyocardium/SUVmaxBone ratio strongly correlated with the presence of low voltage on ECG (r = 0.425, *p* < 0.05). What we currently know is that when low voltage is detected on the electrocardiogram in the presence of myocardial hypertrophy it represents the most specific electrical diagnostic criteria for CA and is an independent predictor for CV death [22,23]. Moreover, in cardiomyopathies, ECG changes precede the clinical onset of the disease [24]. Although it has low sensitivity, its presence is an important indicator of the disease [23]. Considering in many cases a score of 1 results in an equivocal interpretation of the study, it is reasonable to assume that the SUVmaxMyocardium/SUVmaxBone ratio could become a useful quantitative marker in the early diagnosis of TTR cardiac amyloidosis.

A retrospective study by Löfbacka et al. concluded that in patients with ATTRv cardiomyopathy, SPECT-CT parameters DPDmax (equivalent to SUVmax), DPDmean (equivalent to SUVmean) and calculated DPDload indicate amyloid distribution and provides information on cardiac amyloid burden, which correlates with functional cardiac parameters, including GLS [25]. In correspondence to the results of Löfbacka et al., in our study, global longitudinal strain presents a strong correlation with SPECT-CT quantitative parameters SUVmaxMYO/SUVmaxBone (r = −0.495, *p* < 0.05) and SUVmaxMyocadium/SUVmaxLiver (r = −0.692, *p* < 0.05). In current practice GLS has become an alternative to left ventricular ejection fraction to determine systolic function of the heart [26,27]. Recent studies have indicated that no universal cut-off value exists for GLS, as it varies based on factors such as gender and age. [28]. In patient with left ventricular function preserved, GLS provides data related to local and global myocardial deformation, being an excellent marker in the diagnosis of subclinical cardiomyopathies [27,29,30]. Caution is advised when interpreting the GLS data knowing that its modification occurs in many heart diseases (chronic coronary syndrome, valvulopathies, myocardial hypertrophy due to hypertension), therefore it is extremely sensitive in signaling myocardial damage, but non-specific. [31,32].

Another study conducted by Lee et al. analyzed a series of patients with altered GLS suggested that a positive [^99m^Tc]-PYPscan is related to worse segmental, regional and global longitudinal strain LV function as well as diastolic function compared to patients with a negative [^99m^Tc]-PYP result [33]. These findings were corroborated by other studies, which concluded that patients with cardiac amyloidosis exhibit the earliest impairment of GLS compared to other forms of hypertrophic cardiomyopathy. [27,31]. Thus, the presence of the GLS correlation with SUVmaxMyocardium/SUVmaxBone and SUVmaxMyocardium/SUVmaxLiver can be a strong argument in favor of studying the two parameters.

Evaluation at 4 minutes post-gadolinium administration, a subendocardial blood T1 difference of 191 ms detected cardiac amyloidosis with a sensitivity of 90% and specificity of 87% [34]. Because amyloidosis is an infiltrative disease, the extracellular volume is also elevated even when conventional tests and LGE suggest no cardiac involvement, highlighting a potential role of ECV as an early marker of disease [35]. Results from the present studies indicate a very strong positive correlation between gadolinium kinetics/diffusion in the T1 sequence and ECV and SUVmaxMyocardium/SUVmaxLiver (r = 0.814, *p* < 0.05).

A meta-analysis that studied the performance of current imaging methods in the diagnosis of cardiac amyloidosis concluded that SPECT remains the most effective method of evaluation [36]. Knowing that bone-seeking radiotracers have increased avidity for the amyloid deposits of the TTR form of cardiac amyloidosis, SPECT-CT can be considered a method of superior diagnostic value. Future studies comparing ECV with SUVmax Myo/SUVmax Liver can improve the early detection of TTR cardiac amyloidosis.

S II positively correlates with SUVmaxMyocardium/SUVmaxSoftTissue. The potential of this association is highlighted in recent studies, which demonstrate the effectiveness of the S II index in predicting mortality due to cardiovascular events [37] and that we currently know that lung and soft tissue inflammatory activity is significantly higher in those with a Perugini score of 2 and 3 [38]. A recent study in mice shows that the proinflammatory state overexpresses the TTR gene mutation and promotes myocardial amyloid deposition [39].

The absence of correlation with NT proBNP and serum troponin levels was influenced by the presence of mild forms of CA and the administration of heart failure treatment, these findings are consistent with the ones obtained from the study conducted by Löfbacka et al. in which both DPDmean and DPDmax did not correlate with clinical markers. Another possible explanation regarding the absence of a correlation between serum troponin and NT-proBNP levels with the studied parameters could be attributed to the fact that our patient cohort consisted of individuals with hereditary cardiac amyloidosis, who were younger compared to those with wild-type forms. A study by Perfetto et al. demonstrated that NT-proBNP levels are significantly lower in ATTR-CA than in AL-CA amyloidosis, and in ATTRv-CA compared to ATTRwt, despite similar left ventricular mass and renal function. This observation might further explain the lack of correlation between serum biomarkers and SPECT-CT parameters [40]. The use of quantitative SPECT-CT imaging to identify relevant diagnostic parameters such as SUVmaxMyocardium/SUVmaxBone, SUVMyocadium/SUVmaxLiver and SUVmaxMyocardium/SUVmaxSoftTissue for CA evaluation, represent promising advancements in cardiac ATTR evaluation that require dedicated studies in well-defined populations. Selection of a young, large, mutation-carrying population with subclinical forms of the disease, or those with a Perugini score of 1, might be a more effective strategy to establish the role of these quantitative markers in early diagnosis of the disease.

## 5. Limitations

The clinical impact of our findings is currently limited by the relatively small patient sample and the single-center approach; however, correlations that could assist in the early diagnosis of cardiac ATTR were identified.

## 6. Conclusions

The moderate to strong correlations among advanced quantitative SPECT-CT metrics and clinical and paraclinical data create the premises to use these parameters for early diagnosis of cardiac ATTR. The advantages of less interobserver variability by performing more precise parameters than semiquantitative Perugini scores may help for better evaluation of treatment response and prognostication in patients with CA.

Further multicentric studies in a larger patient population are needed to validate the newly identified quantitative SPECT-CT parameters in the diagnosis of patients with suspected ATTR and to advance them from research purposes to clinical application.

## Figures and Tables

**Table 1:** Clinical and imaging parameters of the patients included in the study.

	Gender	Age (Years)	NT_PRO_BNP (Normal Value < 125 pg/mL)	Troponin I (Normal Value < 0.1 ng/mL)	Ecg Low Voltage Features (0 = Absent 1 = Present)	Global Longitudinal Strain—Negative Values (Reference Value −18%)	Myocardial Gadolinium Kinetics with T1 Mapping MRI (0 = Absent 1 = Present)	S II (Normal Value < 335.36)	SIRI (Normal Value < 0.68)	Diastolic Disfunction Severity (*)	Sensory–Motor Polyneuropathy Severity (**)
PATIENT 1	M	67	58.00	0.10	0	16.20	1	497.36	1.06	+	+++
PATIENT 2	F	55	1457.00	0.01	1	9.90	1	773.57	1.84	+++	+++
PATIENT 3	F	48	250.00	0.20	1	14.00	0	2086.55	17.06	+	++
PATIENT 4	M	48	1900.00	0.01	1	9.90	0	297.12	0.65	++	+
PATIENT 5	F	46	3845.00	0.20	1	6.90	0	436.35	1.09	++	+
PATIENT 6	F	46	75.00	0.10	1	19.20	0	451.84	0.34	-	+
PATIENT 7	F	44	136.00	0.01	1	16.80	0	268.16	0.40	+++	+
PATIENT 8	M	36	9.90	0.00	0	17.40	0	49.11	0.14	-	-
PATIENT 9	F	38	30.80	0.35	0	22.00	1	924.86	1.10	-	-
PATIENT 10	F	47	12,977.00	20.60	1	6.80	1	918.99	1.84	+++	+
PATIENT 11	F	58	755.00	13.20	1	12.00	1	445.15	0.55	++	+
PATIENT 12	M	46	939.00	72.3	1	8.00	0	825.76	1.80	+++	+++
PATIENT 13	M	55	47.70	0.20	0	15.80	0	740.57	2.02	+	++
PATIENT 14	M	47	4592.00	0.01	0	8.40	1	1946.16	6.05	+++	+
PATIENT 15	M	47	284.00	2.29	1	12.00	1	502.33	0.20	++	++
PATIENT 16	M	32	4.40	4.76	0	19.00	1	317.85	0.50	-	-
PATIENT 17	M	52	361.00	0.20	0	8.30	1	230.20	2.03	+	-
PATIENT 18	F	53	120.00	0.90	0	16.00	0	416.52	0.70	++	++
PATIENT 19	F	74	300.00	0.10	0	6.80	0	1172.89	0.86	+	+
PATIENT 20	M	86	12,000.00	0.30	1	6.70	0	285.66	1.56	+++	+
PATIENT 21	M	83	250.00	0.10	0	15.80	0	875.32	1.90	+	+
PATIENT 22	F	55	1457.00	0.01	1	9.90	1	773.57	1.84	+	++

0 = ABSENT; 1 = PRESENT. *—normal function (-), impaired relaxation (+), pseudonormal filling (++), restrictive filling (+++). **—asymptomatic (-), mild symptoms (+), moderate symptoms (++), severe symptoms (+++).

**Table 2:** Quantitative SPECT-CT data and heart-to-contralateral (H/CL) ratio.

Heart—Contra Lateral Ratio	SUVmaxMyocardium	SUVmeanMyocardium	SUVmaxLungs	SUVmeanLungs	SUVmaxBone	SUVmeanBone	SUVmaxLiver	SUVmeanLiver	SUVmaxSoft Tissue	SUVmeanSoft Tissue
1.20	3.46	1.07	1.81	0.54	7.83	1.86	2.11	0.49	0.95	0.25
1.52	3.60	2.21	1.77	0.60	5.08	1.92	2.14	0.74	0.91	0.49
1.56	3.44	2.71	2.68	0.36	4.61	1.29	3.44	0.66	0.78	0.34
1.56	2.67	2.28	2.06	0.42	4.63	1.72	2.41	0.61	0.81	0.37
1.43	2.56	2.03	2.11	0.45	4.39	1.49	2.53	0.60	1.47	0.69
1.57	3.82	2.79	2.77	0.57	6.06	1.82	3.62	0.69	1.03	0.60
1.50	4.35	1.94	3.09	0.44	7.86	1.60	3.55	0.48	0.76	0.37
1.12	2.37	2.11	2.19	0.48	10.70	2.24	2.39	0.30	0.79	0.26
0.89	3.10	2.33	3.98	0.55	9.55	2.55	3.20	0.86	0.93	0.35
1.49	5.82	3.54	2.29	0.62	8.43	2.24	2.07	0.99	1.35	0.62
1.66	6.16	4.40	2.66	1.03	6.11	1.75	2.79	1.52	1.37	0.82
1.61	3.93	2.59	2.72	0.59	8.21	1.71	3.53	0.93	1.49	0.84
1.55	3.16	2.50	3.97	0.48	7.09	2.07	3.03	0.80	0.85	0.42
2.02	8.17	5.12	1.88	0.60	9.70	2.27	3.17	1.03	1.14	0.39
1.77	4.58	2.96	2.51	0.63	8.72	2.31	1.71	0.67	1.41	0.58
1.41	4.32	2.33	2.23	0.41	8.90	2.42	3.92	0.75	1.41	0.78
1.69	3.88	1.70	3.52	0.81	9.68	2.57	3.52	1.34	0.43	0.25
1.26	6.15	2.97	2.98	1.01	10.40	2.38	3.61	1.33	1.44	0.65
1.23	3.05	1.41	3.94	0.59	10.30	2.48	2.91	1.04	0.66	0.31
1.38	3.52	1.83	3.15	0.86	10.10	2.07	3.18	1.39	1.71	0.78
1.52	3.43	1.67	2.66	0.62	9.86	2.15	2.14	0.96	0.90	0.40
1.55	1.92	1.05	2.28	0.45	6.89	1.35	2.05	0.63	1.67	0.78

**Table 3:** Quantitative ratios of the SUVmax and SUVmean values of the heart to reference organs (lungs, bone, liver, and soft tissue).

SUVmaxMYO—SUVmaxLungs	SUVmaxMYO—SUVmaxBone	SUVmaxMYO—SUVmaxLiver	SUVmaxMYO—SUVmaxSoft Tissue	SUVmaxMYO —SUVmeanLungs	SUVmaxMYO —SUVmeanBone	SUVmaxMYO —SUVmeanLiver	SUVmaxMYO —SUVmeanSoft Tissue
1.91	0.44	1.64	3.64	6.41	1.86	7.12	13.78
2.03	0.71	1.68	3.96	6.05	1.88	4.89	7.32
1.28	0.75	1.00	4.44	9.64	2.67	5.24	10.15
1.30	0.58	1.11	3.29	6.40	1.55	4.35	7.32
1.21	0.58	1.01	1.74	5.75	1.72	4.25	3.71
1.38	0.63	1.06	3.71	6.67	2.10	5.54	6.41
1.41	0.55	1.23	5.75	9.95	2.72	9.12	11.69
1.08	0.22	0.99	3.00	4.93	1.06	7.93	8.98
0.78	0.32	0.97	3.32	5.64	1.22	3.61	8.96
2.54	0.69	2.81	4.31	9.45	2.60	5.91	9.42
2.32	1.01	2.21	4.50	5.98	3.52	4.05	7.50
1.44	0.48	1.11	2.64	6.62	2.30	4.23	4.69
0.80	0.45	1.04	3.73	6.57	1.53	3.94	7.56
4.35	0.84	2.58	7.17	13.69	3.60	7.93	20.90
1.82	0.53	2.68	3.25	7.28	1.98	6.81	7.86
1.94	0.49	1.10	3.06	10.43	1.79	5.73	5.57
1.10	0.40	1.10	9.11	4.77	1.51	2.90	15.84
2.06	0.59	1.70	4.27	6.09	2.58	4.62	9.51
0.77	0.30	1.05	4.64	5.21	1.23	2.93	9.87
1.12	0.35	1.11	2.06	4.11	1.70	2.53	4.50
1.29	0.35	1.60	3.83	5.50	1.60	3.56	8.58
0.84	0.28	0.94	1.15	4.23	1.42	3.05	2.46

**Table 4:** Correlation between presence/absence of low voltage on ECG and quantitative SUVmaxMyocardium/SUVmaxBone ratio.

		SUVmaxMyocardium/SUVmaxBone
Low voltage ECG	Correlation coefficient	0.425
	Statistical significance	*p* = 0.049

**Table 5:** Correlation between S II and quantitative SUVmaxMyocardium/SUVmaxSoft tissue ratio.

		SUVmaxMyocardium/SUVmaxSoft Tissue
S II	Correlation coefficient	0.556
	Statistical significance	*p* = 0.021

**Table 6:** Correlation between myocardial gadolinium kinetics with T1 mapping MRI and quantitative SPECT-CT parameters.

		SUVmaxMyocardium/SUVmaxLiver
Myocardial gadolinium kinetics with T1 mapping MRI	Correlation coefficient	0.814
	Statistical significance	*p* = 0.049

**Table 7:** Correlation between EchoGLS and SUVmax ratios.

		SUVmaxMyocardium/SUVmaxBone	SUVmaxMyocardium/SUVmaxLiver
EchoGLS	Correlation coefficient	−0.495	−0.692
	Statistical significance	*p* = 0.043	*p* = 0.002

**Table 8:** Correlation between diastolic disfunction severity and quantitative SPECT/CT parameter ratios.

		SUVmaxMyocardium/SUVmaxLiver	SUVmaxMyocardium/SUVmeanBone
Diastolic disfunction	Correlation coefficient	0.486	0.463
	Statistical significance	*p* = 0.022	*p* = 0.030

**Table 9:** Correlation between sensory–motor polyneuropathy severity and quantitative SPECT/CT parameter ratios.

		SUVmaxMyocardium/SUVmaxLiver	SUVmaxMyocardium/SUVmeanBone
Sensory–motor polyneuropathy	Correlation coefficient	0.666	0.450
	Statistical significance	*p* = 0.001	*p* = 0.036

## Data Availability

All data generated or analyzed during this study are included in this manuscript.
